# Nursing factors in performing daily interruption of sedation in a large intensive care unit

**DOI:** 10.1186/2197-425X-3-S1-A323

**Published:** 2015-10-01

**Authors:** L Roberts, C Snelson

**Affiliations:** Department of Critical Care and Anaesthetics, Queen Elizabeth Hospital Birmingham, Birmingham, United Kingdom

## Introduction

Sedation is a fundamental aspect of management of Intensive Care Unit (ICU) patients. Improvements in sedation practice (including daily interruption of sedation [DIS]) have been strongly associated with decreased ICU length of stay and decreased duration of mechanical ventilation [1]. A previous intra-departmental audit had shown scope for improving DIS performance [2]. Nursing care factors impact on efficacious performance of DIS. However, there is limited qualitative evidence determining the specific factors to target in order to optimise patient care.

## Objectives

Identification of modifiable factors within the nursing care provided to ICU patients to allow optimisation of the performance of DIS.

## Methods

This qualitative study was performed in a large 75 bedded ICU with over 450 nursing staff. At the time of study, there was no specific protocol for sedation management within the ICU. Questionnaires were anonymously distributed and collected from all nursing staff members on duty on 6 shifts (day and night). Free text answers underwent qualitative content analysis, coding key themes and concepts. Three specific categories of factors were examined: knowledge, training and clinical practice.

## Results

30% of participants (60; n = 200) responded to the questionnaire. There was a heterogeneous awareness of the benefits of DIS amongst respondents. This was exemplified by 56.7% stating that DIS was an opportunity to assess neurology and 15% noting it as an opportunity to assess the potential for extubation. The most commonly stated disadvantages of DIS were: risk to patient (46.7%), agitation (28.3%) and causing the patient distress (16.7%) - demonstrating similar heterogeneity. The majority (83%) of respondents were unaware of any research concerning DIS. 55.9% were untrained in DIS with 79.3% desiring training/further training. 48.3% stated that doctors led DIS whilst 45% stated that this role was a joint doctor-nurse responsibility. 68.3% felt that it should be led jointly. 61.7% were unaware of the recommended sedation score target during a DIS.

## Conclusions

A number of nursing factors were identified that may be impacting on performance of DSI and patient outcomes. These could largely be rectified with systematic training and education, with the nursing cohort being receptive to this. Introducing a protocol for management of sedation may also help mitigate the human factors impacting on the performance of DSI.
